# Collectanea Medica: Consisting of Anecdotes, Facts, Extracts, Illustrations, &c.

**Published:** 1820-09

**Authors:** 


### COLLECTANEA MEDICA:

CONSISTING OF

### ANECDOTES, FACTS, EXTRACTS, ILLUSTRATIONS, &c.

*Relating to the History or the Art of Medicine, and the Auxiliary Sciences.*

---

Floriferis ut apes in saltibus omnia libant,
Omnia nos itidem depascimur aurea dicta.

---

### *On the Structure of the Membranous Part of the Urethra.* By JOHN SHAW, Esq. Demonstrator of Anatomy, Great Windmill-street.

[From the *Medico-Chirurgical Transactions*, vol. x.]

AN accurate knowledge of the structure of the urethra and bladder is so important to safe practice in the diseases of those parts, that I hope the Society will receive favourably the smallest addition to our knowledge on this subject.

To none are we more indebted than to Mr. Hunter and to Sir Everard Home, for the improvement of this part of surgery; but they, and many surgeons of this country, have described the urethra as a muscular tube; and upon this assumed fact has the practice in stricture been in a great measure founded.

As I have of late years been much in the society of gentlemen who are convinced that the urethra is muscular, I have been induced to pay great attention to the subject, and to examine very particularly the facts adduced by them in proof of its muscularity.

It will perhaps diminish any appearance of presumption in my opposing the opinions of celebrated men, when I inform the Society, that, besides the dissections which I have made for the subject of this paper, and the constant practice I have had in demonstrating these parts, I have, during the last eight years, prepared about seventy specimens of diseases of the urethra and bladder, which are now added to the collection of those diseases in Mr. Bell's museum.

In the course of my dissections, I think I have discovered a peculiar structure in the membranous part of the urethra, which, as far as I can learn, has not been noticed.

But, before describing the structure to which I allude, I may perhaps be allowed to make some observations on the general anatomy of the part, as the decision on the question of the muscularity of the urethra must have much influence in our practice in the treatment of stricture.

It will only be necessary to examine that part of the urethra which is anterior to the insertion of the ejaculator seminis, because the rest of the canal is so surrounded with muscles, that their actions will sufficiently account for all the spasmodic symptoms that occur in the lower part of the passage.

If the anterior part of the urethra be laid open, we see that the inner membrane is continuous with the mucous coat of the bladder; that it is a secreting coat, and has a great many ducts opening upon its surface. If the pudic artery be injected with size and vermilion, the membrane will be seen to be highly vascular; if a portion of the urethra be distended, and the spongy body be carefully removed, the inner membrane will appear delicate and transparent, without the slightest trace of muscular fibres on it. When the urethra is first opened, there is an appearance of muscular fibres running in the length of the canal; but, by examining this with attention, we shall find that it is principally owing to the inner membrane having been thrown into folds by the elasticity of the spongy body. We are referred to comparative anatomy for the ocular demonstration of muscular fibres; and it is confidently asserted, in some late publications, that circular muscular fibres may be seen in the urethra of the horse. I have carefully looked for them in this animal. On a superficial examination, we might suppose that there were muscular fibres in the urethra; but, on farther investigation, the appearance may be proved in this animal, as well as in the human body, to be nothing but the internal membrane thrown into folds by its own elasticity, and by that of the spongy body.*

The venous structure of the spongy body is supported by a set of fibres which have been generally supposed to be muscular, and, if we hold them between the light and the eye, they have certainly some resemblance to muscular fibres; but, if we stretch the part, and again let it go, it will contract. This may be repeated several times, evidently proving that the contractility is not owing to an irritability belonging to the living part, but to elasticity.

I have not been able to discover any fibres in the membrane of the urethra of a man, of a horse, or of an ass; within two inches of the glans of a bull there are a set of fibres which meet at a point, and resemble muscular fibres; but the urethra is possessed of so great a degree of elasticity in this animal, that we must suppose them to be of the same order of fibres as those which are seen in the spongy body.

The muscularity of the urethra has been attempted to be proved more by arguments from analogy than by ocular demonstration; for it is urged, that muscular action does exist, even though muscular fibres be not visible. Of this no one doubts; and I have lately had an opportunity of witnessing the fact. In attempting to inject the lacteals of a turtle, I had great difficulty in pushing on the quicksilver; and, on trying to inject the arteries, I succeeded no better. The cause was apparent when I observed the state of the intestines; for the vermicular motion was still distinct, the viscera having been removed from the animal only a few hours before. On the following day, the viscera having become quite flaccid, the quicksilver was thrown into the lacteals with the greatest ease; and the arteries, which the day before pushed out the injection that had been forcibly driven into

---

* The ejaculator seminis is continued up to the glans in the horse. When we see this strong muscle surrounding the whole of the urethra, we must be at a loss to suggest a use for muscular fibres in the delicate mucous membrane.

them, but did not again contract upon it. This experiment proves that there is more than elasticity in lymphatics and arteries, for the power which prevented the injection from passing into them was destroyed by death; while, in the urethra, we shall find that the power of contraction exists in as great a degree after death as during life.

An hydatid has been given as an example of a body in which there are no muscular fibres visible, but which will contract and dilate to a considerable extent. But is it possible for the muscular power of an hydatid, of a lymphatic, or even of an artery (in which the fibres are visible), to withstand a force equal to that which is frequently used in vain to overcome what is called "spasm of the stricture;" for example, it has been said that "the bougie was grasped with such a degree of firmness, that to withdraw it required a force equal to a pound weight:" but this was an old stricture, five inches and a half below the orifice. The cause of the difficulty of withdrawing a bougie in such a case will be shown presently.

It is said, that, although the muscular fibres may not be seen distinctly in the natural healthy urethra, that they are visible in a canal where there is stricture, and that strictures commence by an irregular action of one of the fibres. There is certainly a fibrous appearance in the urethra posterior to a stricture, but very different from a muscle. It resembles the fibres which are formed on the peritoneum or pleura by inflammation; and in many cases fibres are quite detached from the membrane, except at their two extremities, being similar to the bands which are occasionally seen running across hernial sacs.

If we examine a great number of slight strictures, very few examples of the urethra contracted into a regularly-circular form will be found; but, in the greater number of cases, about one-half or two-thirds of a portion of the urethra will have become white and dense, or a firm white line will be seen running obliquely along the passage; and yet we do not find any oblique fibres described in the natural state of the parts. We must account for the formation of stricture on other grounds than the contraction of muscular fibres; for we find, by dissection, appearances which denote previous inflammation. There is a white dense line, not pliant like the other parts of the urethra, but firm and exactly similar to the stricture formed in the inner coat of the œsophagus, or to a thickening of a portion of the pleura or peritoneum.

The changes which are supposed to take place in the size of the canal during the natural actions, and the phenomena of disease, have been both adduced as arguments in favour of the muscularity of the urethra; for example, the diminution of the canal during the emission of the semen is given as a proof of muscularity; but the same diminution may be produced after death, by injecting the spongy body. The expulsion of an injection in a case of gonorrhea has been considered as one of the most decided proofs; but, if we throw an injection into the dead penis, the fluid will be thrown out with as much force as in the living body. I threw a small quantity of water into the corpus spongiosum, so as to swell the penis a little, making it resemble the state in which we generally see it in gonorrhea. On injecting the urethra, the fluid was thrown out nearly two yards. Surely no one will say that there was muscular action existing in the urethra of a body almost putrid; but, on the contrary, it will be allowed that the injection was expelled by the elasticity of the parts. That this elasticity is as perfect after death as during life, may be proved by the examination of the urethra of a horse; for the canal, though very small, will allow of dilatation sufficient for the admission of the thumb; and, on withdrawing the thumb, the urethra will regain its former size: and this may be repeated several times. Is there any muscular part which, after death, would admit of such a degree of dilatation, and again contract to its former size?

The expulsion of a bougie by the natural actions of the urethra, has been given as another proof; but this may be also met by an experiment on the dead body. If we distend the spongy body slightly, and then introduce a bougie into the urethra, it will be gradually pushed out. We must recollect that, when a large bougie is passed in the living body, the penis is pulled up upon it. When we let the parts go, the penis recedes from the bougie, the muscles push it out a certain extent, and then there remains so small a portion, that we can readily conceive the elasticity of the parts to be sufficient to press out the remainder of this *conical* instrument.

The question is asked, " why does the urine flow in a diminished stream during an attack of gonorrhœa?" While the inflammatory stage continues, (at which time alone is there difficulty of passing the urine,) the calibre of the canal is, to a certain extent, diminished by the swollen state of the parts; but, what is of more consequence, the muscles of the perineum and the detrusor urinæ are very irregular in their actions, in consequence of the increased sensibility of the membrane to the acrid urine; and, if we recollect the numerous muscles which surround the lower part of the canal, we can have no difficulty in explaining why the urine should occasionally stop, or why a stimulating injection should be prevented from going into the bladder.

We find the following expression very commonly made use of. " Spasm comes on a stricture so as to prevent the passage of a bougie, which a short time before entered freely." We must acknowledge that there is a sensation which gives this idea very frequently experienced in the lower part of the canal. To explain this symptom, it will be necessary to refer to the consideration of the natural action of the muscles of the bladder, and those surrounding the urethra. The bladder is furnished with a strong muscle, the detrusor urinæ, and the lower part of the urethra is surrounded by a set of muscles which may be considered by their actions as the sphincters of the bladder. Before the urine can flow, it is necessary that the detrusor urinæ should contract, and that the muscles surrounding the urethra should at the same time relax. While a person is in perfect health, it is not possible to make the urine flow by pressure on the abdomen; but, in a paralytic state of the lower part of the body, the urine may be forced out by pressure, because in this state the muscles of the perineum are passive, while, by pressure, the action of the detrusor is imitated. I have witnessed a case which gave a very striking proof of this.

A gentleman with stricture had been so entirely mismanaged, that the bladder was allowed to become much distended, and, being neglected in this state, he became comatose. It was not possible to introduce a catheter. Instead of puncturing the bladder, which could have been the only hope in such a case, the surgeon was satisfied with trying to empty the bladder by pressing on the abdomen. As the patient was now paralytic, the urine flowed through the narrow stricture; upon which the surgeon exultingly said, "You see there is no necessity for doing any thing more." The same effect would have been produced by pressure on the distended bladder of the dead body. Thus, a certain sympathy must exist between the two sets of muscles before the natural action can take place; but this sympathy is very apt to be disturbed by any cause producing irritation on the inner membrane of the urethra, and particularly by stricture; for the point of stricture is always the seat of irritation. The urine is pressed forward by the detrusor urinæ, the muscles of the perineum relax, until the urine comes up to the stricture; but there the acrid urine naturally produces irritation, and the surrounding muscles are called into action, as they are governed by the sensibility of the membrane. For the same reason, the muscles are irritated so as to prevent the farther entry of a bougie, which has been passed down to an irritable stricture. The means of removing this spasm give us the best idea of its cause. By passing down the caustic to an irritable stricture, the morbid sensibility of the stricture is destroyed, and then a bougie will pass, or the urine will flow, without producing such an excitement as will bring on spasm of the surrounding muscles, which, when present, has been called "spasm of the stricture."*

This explanation will not account for spasm which is alleged to take place in the canal anterior to the insertion of the ejaculator seminis. I have considerable difficulty in arguing this part of the question, because I have never seen a spasm of the urethra at the glans, and I have been assured by surgeons of great eminence that they have never seen it; yet I know that there are some surgeons who say that, by a single touch of the caustic, they have seen the urethra at the glans fly open, which the minute before was spasmodically contracted. Instead of insisting that the invisible muscular fibres form the obstacle to the passage of a bougie in the anterior part of the urethra, may we not rather assign some of the following causes? On introducing a bougie, the point may at first strike against the edge of the stricture, but, becoming softened, it may turn up and pass through; it may have got into one of the lacunæ, or, by exciting irritation, it may produce a sudden flow of blood into the corpus spongiosum, which will gradually subside, and then the bougie will pass easily; for we may see by an experiment, that the slightest quantity of water thrown into the veins of the spongy body diminishes the calibre of the urethra very much.

* Antispasmodic medicines relieve the spasm by subduing the irritation. It is necessary to remark, that the cases which are recorded as examples of relief from the use of those medicines, were strictures in the lower part of the urethra, and were completely within the action of the strong muscles.

The following symptom of spasm may take place at any part of the canal. "The bougie is grasped and held by a spasm in a stricture." I have seen a gentleman tug at a catgut bougie, which he had passed through a very old narrow stricture, and heard him say, "Mark what a degree of spasm there is." On withdrawing the bougie, it was found that the part which had been passed through the stricture had become soft and swollen, while the part which was embraced by the stricture was firm and round. In attempting to withdraw the bougie, he had pulled the button-like end of the instrument against the firm stricture. The same thing happens, in a slighter degree, to the wax bougie. The instrument is pushed with a certain force through the stricture; by lying in the passage, it becomes softened; that part which is in immediate contact with the stricture is indented; for, though the stricture was forcibly distended by the bougie while hard, it will make an impression on the softened instrument. But it is curious that a stricture in almost a cartilaginous state should be supposed to be capable of contraction and dilatation. Do we find any thing analogous to this in the muscles; does the portion of the sterno-cleido-mastoideus, which, when cartilaginous, produces the wry-neck, ever contract or elongate?

Were the urethra muscular, would it not throw out the gonorrhœal matter with a jerk; but can a patient ever do that? Were it muscular, in a state of disease, should we not find it contracted and fibrous, as the bladder is in a case of stricture? To prove that it is not so, I shall describe only one of the numerous examples which are to be seen in Mr. Bell's museum. There is a stricture near the glans which admits only a bristle. All the urethra behind is so enlarged, that the finger may lie in any part of it, and, instead of its surface being fibrous, it is perfectly smooth, while the bladder is strong and muscular. If we allow the urethra to be a passive membrane, we can then understand how it has become so enlarged by the continued action of the detrusor urinæ to throw the urine through the stricture. But it may be asked, why is not the canal collapsed by its own elasticity, and by the pressure of the elastic spongy body? In answer to this, we find that the spongy body, in the greater number of bad strictures, becomes quite altered in structure by the continued irritation; the oblique fibres adhere together, and in many of these cases the whole penis wastes, is not capable of erection, and becomes wonderfully short and small; while in others, apparently by a deposition of lymph during the attacks of inflammation, the penis is permanently enlarged.

Is it possible for a muscle, which, if it does exist, is allowed by all to be of a finer structure than any muscle of the body, to resist the action of the fibres of the bladder, which, in cases of stricture, are so strong? or can it resist the weight of the hand, and such force as is sometimes used to overcome the "spasm?" Is it possible to preserve in spirits a spasm of a muscle, after the part has been macerated almost to putrefaction?* With as much truth may we call the preparations

* There may be seen in anatomical museums preparations marked "Specimen of Spasmodic Stricture."

of the thickened membrane in hernial sacs examples of the spasmodic affection of the peritoneum.

I shall now describe that structure to which I referred in the beginning of the paper, and which, as an anatomical fact, should go a great way to settle the question of the muscularity of the urethra.

The membranous part of the urethra has been always described as that portion which is between the prostate and the bulb, and is called membranous, to distinguish it from those parts of the canal which are surrounded by the prostate gland or the corpus spongiosum.

The preparations and drawings which are upon the table will show that there is a structure within the membranous part which in some degree resembles the spongy body. It may be seen in the horse, as distinct as the rete vasculosum in the vagina, by merely dividing the urethra; but it is necessary to inject it, before we can show it in the human body. It may be injected in several different ways: if we put the injecting-pipe into the spongy body, by using a very little pressure, we may fill the corpus spongiosum from the bulb to the glans; if we use more force, and then cut through the cavernous body, so as to expose the lower surface of the urethra, we shall probably see a regular vascularstructure immediately under the mucous membrane, which does not terminate at the bulb of the corpus spongiosum, but passes into the membranous part.

The best manner of showing this structure, is to puncture the mucous membrane, and to introduce the mercurial injecting-tube under it. If the pipe be properly introduced, the mercury will not flow into the spongy body, but will pass under the membrane of the urethra, filling a very remarkable net-work of veins. These veins are spread all over the urethra, but at the membranous part they are accumulated, lying one over another in the length of the canal, so as to form two distinct columns, with a groove between them, which extends from the caput gallinaginis to the glans. The columns unite and surround the membrane forming the sinus pocularis, which itself, from its vascularity, appears to be capable of erection. The peculiar character of the vascular structure is lost on the prostate, by the termination of the vessels in a net-work of veins, which have communication with the common veins of the bladder. If we continue the injecting force, and interrupt the course of the mercury, it will pass into the spongy body, proving that there is a communication between the corpus spongiosum and this vascular structure, but by no means so free as between the glans and the bulb.*

---

* I had by chance filled some of those vessels in the upper part of the urethra, some years ago, while preparing the parts for demonstration. I was rather checked in the pleasure of thinking I had made a discovery, on being told by Mr. Bell that the same vessels had been described by Dr. Barclay. On referring to Dr. Barclay's paper, in the first volume of the *Edinburgh Medical and Surgical Journal*, I found that he had only filled a few of those vessels on the upper part of the urethra, which, by some anatomists, had been supposed to be lymphatics. I pursued my investigation, and discovered the spongy body surrounding the membranous part. Since I wrote this paper, I have seen the work of Professor Moreschi, of Milan, *Commentarium de Urethræ Corporis, Glandisque Structura;*

I have placed upon the table the bladder and lower part of the urethra of a stallion pony. There are many points of the anatomy of the human urethra explained by this preparation. The two columns and the channel between them are more distinctly seen than in man, because the corpus spongiosum in the horse is more evidently divided into two portions. It is so completely separated on the upper part, that about an inch of the urethra near the glans is not, on its lower surface, covered by the spongy body. It is very easy to separate, in this animal, the bulb from the internal spongy body; in the preparation on the table it is held suspended from the internal spongy body, which is seen to pass along the membranous part of the urethra in two columns, that unite and form another bulb anterior to the prostate.*

This structure in the human body is of much importance in a surgical point of view; for, when we consider its great vascularity, we cannot be surprised that the vessels should be so often opened by our instruments, and continue to throw out blood for a length of time, if excited by a degree of erection. Perhaps the cases where the patient has been so miserable, from defective action in those parts where the semen is passed with the urine, may admit of explanation, by the facts which have been detailed; for, in the greater number of the instances where the semen was pressed back into the bladder, there has been an imperfect erection, and, consequently, the little eminence, which may be called the internal bulb, would not be distended so as to fill the urethra, and direct the semen forwards into the channel, between the two columns, while the ejaculator seminis was acting spasmodically upon it in the sinus.

I may be now permitted to take this structure as an anatomical fact, to disprove the existence of muscular fibres in any part of the canal; for I have been able to show, that what has been described as muscular fibres immediately under the mucous membrane, is the uninjected vascular texture of the internal spongy body.

---

and I have also had a conversation with Professor Antomarchi, of Florence, the Editor of Mascagni's posthumous works; by which I find, that the researches of the Italian anatomists had not extended to the membranous part of the urethra, but had been confined to the upper part, to determine the question so much agitated upon the Continent at present, whether the structure of the corpora cavernosa and spongiosa be vascular or cellular?

* Cowper's glands and the prostate are preserved in this preparation; they form an excellent illustration of the structure of these parts in the human subject. The vesicula seminalis in the horse is really a bladder, and is surrounded with muscular fibres nearly as strong as those of the urinary bladder.

